# Validity and Reliability of the Digital Functioning Assessment Short Test (D-FAST) in the Brazilian Sample

**DOI:** 10.2174/17450179-v18-e2210121

**Published:** 2022-10-19

**Authors:** Silvia Dubou Serafim, Jeferson Ferraz Goularte, Marco Antonio Caldieraro, Flavia Moreira Lima, Giovana Dalpiaz, Francisco Diego Rabelo-da-Ponte, Carla Torrent, Brisa Solé, Eduard Vieta, Adriane Ribeiro Rosa

**Affiliations:** 1 Laboratory of Molecular Psychiatry, Hospital de Clínicas de Porto Alegre, University Federal of Rio Grande do Sul, Porto Alegre, Rio Grande do Sul, RS, Brazil; 2 Postgraduate Program in Psychiatry and Behavioral Sciences, University Federal of Rio Grande do Sul, Porto Alegre, RS, Brazil; 3 Hospital de Clínicas de Porto Alegre, University Federal of Rio Grande do Sul , Porto Alegre, Rio Grande do Sul, RS, Brazil; 4 Bipolar and Depressive Disorders Unit, Hospital Clinic, University of Barcelona, Institute of Neuroscience, IDIBAPS, CIBERSAM, c/Villarroel, 170, 12-0, 08036 Barcelona, Catalonia, Spain; 5 Departament of Pharmacology, Institute of Basic Health Sciences, Federal University of Rio Grande do Sul, Porto Alegre, RS, Brazil

**Keywords:** COVID-19, Pandemic, Public health, Deaths, Virus transmission, Psychosocial implications

## Abstract

**Background:**

The COVID-19 pandemic has caused major disruptions in all aspects of daily functioning, from school and work to interactions with friends and family. The Functioning Assessment Short Test (FAST) is an interviewer-administered scale validated in the psychiatric sample with no previous study assessing its validity and reliability in a digital format. Thus, we aimed to analyse the psychometric properties of the digital version of the FAST and understand the implications of COVID-19 and restrictive measures on functioning.

**Methods:**

Data were collected using an online survey. The psychometric properties of the digital FAST were assessed by confirmatory factor analysis, Cronbach’s alpha, and discriminant functional by cluster analysis in a community sample.

**Results:**

Out of the total sample, 2,543 (84.1%) were female, and the mean (SD) age was 34.28 (12.46) years. The digital FAST retained the six factors structure observed in the original version, with Cronbach’s alpha above 0.9. In addition, we showed evidence of discriminant validity by differentiating three clusters of psychosocial functioning. Clinical and demographic differences between groups explained, in part, the heterogeneity of functioning, thus providing support for the construct validity of the instrument.

**Conclusion:**

The digital FAST is a simple and easy-to-understand instrument that provides a multidimensional assessment of functioning without the need for an interviewer. Furthermore, our findings may help to better understand the psychosocial implications of the pandemic and the importance of planning specific interventions to rehabilitee the affected group.

## 1 INTRODUCTION

On January 30^th^, 2020, the World Health Organization (WHO) announced that severe acute respiratory syndrome coronavirus (COVID-19) was a public health emergency of international concern. Currently, COVID-19 has infected over 125 million people and resulted in over two million deaths worldwide, while in Brazil, it has infected over 21 million people and resulted in over 610 thousand deaths (as of November 13^th^, 2021) [1]. According to the WHO, respiratory etiquette, hand washing, and physical distancing must be maintained to control virus transmission, while most people have not been vaccinated. However, the pandemic and restrictive measures may be particularly harmful to low-income and middle-income countries in which weak social safety nets and economic resources are not sufficient to cover daily needs [2], such as Brazil, where the greatest risk of disease trans-mission is among the poorest communities [3].

The pandemic and containment measures [4] have changed daily routines, bringing several physical and mental consequences, such as emotional distress and increased risk of psychiatric illnesses, especially among the most vulnerable groups [5]. Indeed, mental health burdens and increased use of mental health services are expected as a consequence of the pandemic. The high prevalence of anxiety, depression, and other stress-related disorders observed during the acute phase of COVID-19 has been consistently reported in multicultural studies [6, 5, 7-9]. Notably, these psychiatric conditions are among the leading contributors to disability worldwide [10]. Thus, the relationship between mental health and functional impairment is not new in psychiatry.

Psychosocial functioning describes a person’s ability to perform the tasks of everyday life, engage in relationships with others in ways that are gratifying to the individual and others, and meet the needs of the community in which the person lives [11]. The COVID-19 pandemic caused major disruptions in all aspects of functioning, from school and work to interactions with friends or family and recreational time. Furthermore, the measures to mitigate the disease have substantially altered the economic scenario with an increase in unemployment and uncertainty about the future [12], which contribute to the worsening of mental health. Thus, a better understanding of the pattern of psychosocial functioning among general populations during the COVID-19 pandemic would be of clinical utility as such information could contribute to the development of interventions focused on functional restoration.

The Functioning Assessment Short Test (FAST) [13] is an interviewer-administered scale involving the use of pen and paper that allows the multidimensional evaluation of functioning, including aspects, such as the individual’s ability to function socially or occupationally or to live independently as well as financial issues and cognition. The FAST scale was originally validated in several languages in distinct clinical samples [13-21] as well as in healthy individuals [22, 23], with no previous study assessing its validity in self-reported digital format. The issue with transferring such validated instruments to the digital format has been raised by some authors [24], with others suggesting that a validated pen and paper scale needs further validation when used in other formats, including online assessments [25]. In addition, although many researchers have assessed the impacts of COVID-19 on mental health [6, 5, 7-9], no data are yet available on the effects of the current pandemic on psychosocial functioning.

Hence, the purpose of the present study was to analyze the psychometric properties of the online self-reported version of the FAST and also to understand the implications of COVID-19 and restrictive measures on psychosocial functioning by cluster analyses in a subsample of the Brazilian population during the first wave of coronavirus transmission.

## 2 MATERIALS AND METHODS

### 2.1 Participants

A cross-sectional web-based survey was carried out using an anonymous online questionnaire distributed via social networks. The data were collected from May 20^th^ to September 13^th^, 2020, during the first peak of COVID-19 in Brazil. The online questionnaire consisted of sociodemographic variables and the assessment of psychosocial functioning, physical, and mental health status, including the history of previous psychiatric disorders and the severity of stress, anxiety, and depression as described below. The inclusion criteria included being older than 18 and living in Brazil at the time of the survey. All participants provided online informed consent. The local ethical committees approved all procedures.

### 2.2 Assessments

a) Psychosocial functioning was assessed by the Functioning Assessment Short Test (FAST) scale [13], which allows for the evaluation of the main aspects of functioning: autonomy, occupational functioning, cognitive functioning, interpersonal relationships, financial issues, and leisure time. All items of scale are rated using a four-point Likert scale: 0 = no difficulty, 1 = mild difficulty, 2 = moderate difficulty, and 3 = severe difficulty. The global score is the sum of all items. The higher the score, the more severe the difficulties. The FAST is an interviewer-administered, transdiagnostic scale validated in distinct clinical samples and available in several languages [13-21]. It was also validated in healthy individuals [22, 23]. In this web-based survey, the online self-reported version of the FAST was used for the first time; a brief description of each item was included in this version in order to guarantee the best understanding by the responders (supplementary material).

b) The Impact of Event Scale-Revised (IES-R) is a self-rated 22-item questionnaire divided into three domains (avoidance, intrusion, and hyperarousal), which evaluates the degree of distress caused by a traumatic event [26]. Each item is rated on a five-point Likert scale (0 = not at all; 1 = a little bit; 2 = moderately; 3 = quite a bit; 4 = extremely). The IES-R total score is the sum of the average of each domain. A total score higher than 5.6 indicates psychological stress.

c) The Patient-Reported Outcomes Measurement Information System (PROMIS) for depression (PROMIS Short Form v1.0 - Depression 8a) assesses negative mood (sadness, guilt), views of self (self-criticism, worthlessness), social cognition (loneliness, interpersonal alienation), and decreased positive affect and engagement (loss of interest, meaning, and purpose).

d) The PROMIS anxiety assesses self-reported fear (fearfulness, panic), anxious misery (worry, dread), hyperarousal (tension, nervousness, restlessness), and somatic symptoms related to arousal (racing heart, dizziness).

Both PROMIS instruments consist of an eight-item questionnaire that assesses symptoms over the period of seven days, with items rated on a five-point Likert scale (1 = never; 2 = rarely; 3 = sometimes; 4 = often; 5 = always). All PROMIS scores were presented as T-scores calculated by the Health Measures Scoring Service (https://www.assessmentcenter .net/ac_scoringservice) from the raw sum score, using T-scores from the United States general population. The T-score is the standardized score with a mean of 50 and a standard deviation of 10. For depression and anxiety, T-scores lower or equal to 55 indicate no significant symptoms, higher than 55 to 60 indicate mild symptoms, higher than 60 to 70 indicate moderate symptoms, and higher than 70 to 83.1 and 81.1, respectively, indicate severe symptoms.

## 3 STATISTICAL ANALYSIS

### 3.1 Confirmatory Factor Analysis

R (version 4.0.2) and RStudio (version 3.5.3) software were used for all analyses. We applied a confirmatory factor analysis (CFA) to identify the factorial structures of a set of items. Furthermore, CFA is highly useful in verifying the relationship between observed variables and latent traits. We performed a CFA through the principal axis factoring method in order to describe the internal structure of the online self-reported FAST scale and to confirm the same factors found in the pen and paper version of FAST using the package “lavaan” (version 0.6-12). Then, we used the oblimin rotation with the Kaiser-Meyer-Olkin (KMO) measure of sampling adequacy (>0.5) and Bartlett's Test of Sphericity (p<0.05) to confirm whether those metrics met all assumptions for CFA [27]. Afterward, we investigated all eigenvalues over Kaiser’s criterion of 1 to confirm the number of factors and the number of items for each factor.

### 3.2 Internal Reliability

The Cronbach’s alpha was used to analyze the internal reliability of the online FAST global factor (*i.e*., FAST total score) and was assessed using the following criteria: α ≤ 0.9, excellent; α ≤ 0.8, good; α ≤ 0.7, adequate (package “ltm”, version 1.2-0) [28]. Cronbach’s alpha is a coefficient of reliability among raters; in other words, a high value for Chronbach's alpha means all the psychometric items measure the same construct.

### 3.3 Discriminant Validity

All functioning domains were converted into Z-score using all samples. Afterward, we performed the Partition Around Medoids (PAM) algorithm [29] to identify functioning clusters of subjects during the COVID-19 outbreak (package fpc, version 2.2-9) as a proxy of discriminant validity. PAM algorithm was used rather than k-means, a classical clustering algorithm, because it is more robust to noise and outliers, minimizing the sum of dissimilarities between data points. To calculate the dissimilarities between pairs of subjects, Gower’s distance was applied. The optimal number of clusters was determined by the Gap statistic method using the package “factoextra” (version 1.0.7). After the clustering, a discriminant function analysis (DFA) was performed using the package “MASS” (version 7.3-51.6) to confirm the clusters retained and investigate the predictive power of the clustering of each individual’s functioning domain to the functioning subgroup.

### 3.4 Univariate and Multivariate Analysis

Furthermore, we conducted comparisons (one-way ANOVA with Tukey HSD post-hoc test and χ2 applied as appropriate) between the different clusters to examine possible differences in functional status, demographic and clinical variables. Moreover, multinomial logistic regression (package “nnet,” version 7.3-14) was carried out using the good functioning cluster as the reference category outcome in order to identify predictors of functioning. The model included the following variables: age, T-score of depression, T-score of anxiety, gender, work status, household income, work status in the ongoing pandemic, previous history of psychiatric illness, marital status, education, and post-traumatic stress. We also calculated the effect size using Hedges’ g from the mean and standard deviation between good functioning vs. intermediate functioning and good functioning vs. low functioning (package “esc,” version 0.5.1). Positive values for Hedges’ g mean a good functioning cluster that shows better performance than intermediate or poor functioning. Statistical significance was set at p < .05.

## 4 RESULTS

### 4.1 Demographic Characteristics

A total of 3,023 individuals completed the survey. Out of the total sample, 2,543 (84.1%) were female, and the mean (SD) age was 34.28 (12.46) years.

### 4.2 Confirmatory Factor Analysis

The original version of FAST is based on a six-factor structure [13]. In this study, the online self-reported FAST was assessed for fitting the same structure. Initially, the Kaiser–Meyer–Olkin test verified the sampling adequacy for the analysis (KMO = 0.91) with good values for Bartlett’s test for sphericity (χ^2^(276) = 32,288, p < 0.001). In addition, an initial analysis was performed to obtain eigenvalues for each factor in the data. In agreement with the original FAST, the online FAST showed that six factors had eigenvalues over Kaiser’s criterion of 1 and explained 63.08% of the variance in combination. We retained six factors due to the large sample size and the convergence of the scree plot and Kaiser’s criterion. The same internal structure (*i.e*., six factors) of the original version was also reported in the online self-reported FAST except for two items, *i.e*., “participating in social activities” and “having satisfactory sexual relationships,” shifting from the interpersonal relationships factor to the leisure time factor. However, as leisure time and interpersonal relationships were strongly related, this change did not compromise the internal structure of the online FAST. Hence, the maximum score of the interpersonal relationships and leisure time domains was 12 in the online version compared to scores of 18 and 6, respectively, as described in the original scale. The internal consistency coefficient presented an overall Cronbach = 0.95, indicating excellent internal reliability of the online FAST.

### 4.3 Discriminant Validity

The PAM algorithm, through the Gap statistic method, provided evidence for three functional clusters among the 3,023 volunteers, as demonstrated in Fig. (**1**). The first cluster included 661 subjects (21.86%) who presented low functioning (LF). The second one contained 1,436 subjects (47.50%) with intermediate functioning results (IF). The last cluster had 926 patients (30.36%) with good functioning (GF). The discriminant function analysis (DFA) exhibited two discriminant functions, which explained 99.4% and 0.6% of the variance, respectively (Wilks’ λ = 0.19, χ2 (12) = 4884.41, p< 0.001; Wilks’ λ = 0.97, χ2 (5)= 65.1, p < 0.001). The subjects were classified by DFA into 89.2% of the cases, demonstrating the validity of the three functioning clusters. The cognitive domain and leisure time domain were among the domains that better classified participants into functioning clusters (Function 1: r = 0.46 and r = -0.37; function 2: r = 0.4 and r = 0.67, respectively) Fig. (**2**) for graphical agglomeration of the functional subgroups).

The LF group showed marked impairment in all FAST subdomains, with a huge difference in mean scores between LF and GF. The IF cluster exhibited an intermediate level of functioning in all subdomains, with a great difference in mean scores between IF and GF. Finally, the GF cluster presented a high-functioning performance in all subdomains (Table **1**). Taken together, these data suggested that the online self-reported FAST scale could discriminate subjects with different levels of psychosocial functioning, thus supporting the discriminant validity.

### 4.4 Variables Potentially Affecting the Overall FAST Score

As shown in Table **2**, concerning sociodemographic variables, the one-way ANOVA with Tukey HSD post-hoc test and χ2 showed differences among the three functioning groups in all characteristics.

As shown in Table **2**, concerning sociodemographic variables, the one-way ANOVA with Tukey HSD post-hoc test and χ2 showed differences among the three functioning groups in all characteristics.

In the multinomial regression analysis, the model showed a good fit to the data (Deviance: χ^2^ = 4756,724, *df* = 5968, p = 1.00; Nagelkerke’s *R^2^* = 0.455) and was significant to account for variance in the cluster functioning (Model χ^2^ (56) = 1529,46, p < 0.001). The multinomial regression showed that higher scores in PROMIS depression (OR = 1.21(1.18 – 1.24) 95%CI, p < 0.001) and PROMIS anxiety (OR = 1.05, 95%CI (1.03 to 1.08), p < 0.001) significantly predicted whether the responder belongs to the low functioning cluster or the good functioning cluster, with a high relative risk belonging to the low functioning cluster. Furthermore, lower (OR = 3.42, 95%CI (2.18 to 5.35), p < 0.001) and middle (OR = 1.65, 95%CI (1.09 to 2.49), p < 0.01) household income significantly predicted whether the responder belongs to the low functioning cluster or the good functioning cluster, with higher odds belonging to the low functioning cluster. On the other hand, no previous history of psychiatric disorder (OR = 0.53, 95%CI (0.40 to 0.34), p < 0.001) and no symptoms of post-traumatic stress disorder (OR: 0.24, 95%CI (0.18 to 0.34), p < 0.001) significantly predicted whether the responder belongs to the low or the good functioning cluster, with a lower odd belonging to the low functioning cluster.

Furthermore, multinomial regression showed that younger age (OR: 0.99, 95%CI (0.98 to 1.00), p < 0.05), higher PROMIS depression (OR: 1.09, 95%CI (1.08 to 1.11), p < 0.001), and PROMIS anxiety scores (OR: 1.04, 95%CI (1.01 to 1.05), p < 0.001) significantly predicted whether the responder belongs to the intermediate or the good functioning cluster, with a high relative risk belonging to the intermediate functioning cluster. Moreover, lower (OR: 1.87, 95%CI 1.39 to 2.51, p < 0.001) and middle household income (OR: 1.40, 95%CI 1.10 to 1.79, p < 0.01) significantly predicted whether the responder belongs to the intermediate functioning cluster or the good functioning cluster, with higher odds belonging to the intermediate functioning cluster. However, lower education (OR = 0.72, 95%CI 0.58 to 0.89, p < 0.01) and no symptoms of post-traumatic stress disorder (OR = 0.58, 95%CI 0.44 to 0.77, p < 0.001) significantly predicted whether the responder belongs to the intermediate or the good functioning cluster, with a lower odds belonging to the intermediate functioning cluster.

## 5 DISCUSSION

The present study evaluated the psychometric properties of the online self-reported FAST scale and psychosocial implications of COVID-19 in a general population during the first peak of SARS-CoV-2 transmission in Brazil. The results showed that the online FAST scale retained the same six domains as the original version, and the items had high internal consistency, with Cronbach's alpha above 0.9. In addition, the online version showed evidence of discriminant validity by differentiating three categories of psychosocial functioning in the sample and related variables with an overall FAST score. Our results showed the applicability of the digital FAST scale to assess functioning in the general population and revealed the main predictors of functional impairment during the first wave of SARS-CoV-2 contagion.

The ongoing pandemic imposes barriers for researchers worldwide, with many studies using online surveys to assess mental health in the general population [6, 30]. However, many instruments used in online studies have not been previously validated in the digital format and do not always reproduce the psychometric properties of original versions of the scales [31-33]. For instance, the assessment of anxiety in patients with panic disorder by the internet-based Beck Anxiety Inventory (BDI) questionnaire showed a significant difference in mean scores, with lower scores observed in the internet version compared to the original version of the scale [30]. In the present study, the internal consistency and reliability were found to be similar to a study that assessed the psychometric properties of the older version in bipolar patients and healthy controls by establishing a six-factor internal structure and a Cronbach’s alpha of 0.9109 [13]. Furthermore, the psychometric properties of FAST in the present study were similar to the findings reported in a study on a subsample of adults with Autism Spectrum Disorder (ASD) or a sample involving patients in first-episode psychosis that also showed a six-factor structure and a Cronbach’s alpha of 0.91 and 0.88, respectively [14, 18]. In addition, analysis of FAST reliability and factorial structure performed in patients with first-episode psychosis and healthy controls showed adequate reliability (Cronbach’s alpha of 0.882) and a six-factor structure, suggesting that the FAST scale is applicable to a range of health conditions. However, mean scores of a high-functioning cluster were slightly higher than the cut-off observed in the older version [13], suggesting that misclassification of functional status would occur in the web survey if we applied a cut-off of the older version of FAST. Therefore, studies assessing the reliability and validity of new digital instruments, even those shifting from pen and paper format, are now required as many web surveys are in progress. In addition to reliability, the self-reported online FAST discriminated subjects into three clusters of functioning as follows: (I) good functioning group, representing one-third of individuals that experienced satisfactory functioning in distinct life domains, (II) intermediate functioning group, representing almost 50% of individuals with mild deficits in domains of functioning, and (III) low functioning group representing around 20% of individuals that experienced global and significant impairment in all domains of functioning. Some variables, namely age, income, mental health, and history of psychiatric disorder, significantly explain these functioning clusters. Responders in the LF cluster experienced greater anxiety and depressive symptoms as they self-reported more past events related to psychiatric disorders than other groups; therefore, both factors correlated to poor outcomes. Indeed, psychiatric symptoms and cognitive deficits have been traditionally associated with a higher overall FAST score or poor functioning in clinical samples [13, 34].

The LF cluster also had lower socioeconomic levels and more financial difficulties than the other two clusters. Indeed, impairment in occupational functioning and financial issues might be a consequence of COVID-19 and pandemic preventive measures since there was an increase in unemployment rates, thus leading to more financial strain [35, 36]. The economic impact of the pandemic may aggravate the condition of more vulnerable individuals that, unfortunately, represent a huge part of the population in developing countries, like Brazil. Finally, younger people that were more prevalent in LF than other groups also reported more negative effects of COVID-19 than older subjects. Probably, pre-pandemic distress, such as educational, professional, or social difficulties typically experienced by young adults, compounded by lifestyle disruptions and feelings of hopelessness during the pandemic, may have contributed to these findings [37]. Additionally, they are more vulnerable to stressful situations because of their inexperience and lack of adaptative mechanisms. Together, these findings support that the digital FAST scale was sensitive to detect differences in functioning in a large sample of discriminating individuals by clinical symptoms and demographic characteristics, highlighting the potential utility of this scale in clinical and research settings.

One of the strengths of this instrument is that it is being validated in several cultures in either clinical samples or the general population. The self-reported online version showed strong psychometric properties, which are quite similar to its original version. The FAST may contribute to a multidimensional assessment of functioning with the advantage of being one of the very few validated scales in a digital format in a large community sample.

Nevertheless, some important limitations should be mentioned. Firstly, we used an online survey with a convenience sample method. Secondly, the instruments used to assess mental health were self-reported and might not characterize mental health status with the accuracy of structured clinical interviews. Also, although the digital version of the FAST showed satisfactory psychometric properties, we could not compare scores between different formats of the instrument to assess intra-class coefficients and mean scores; both measures are used to evaluate the degree of reliability and equivalence of the same instrument delivered using different formats [38]. Finally, it is noteworthy that online survey methods may have an issue of biased sampling toward people with good internet literacy and access.

## CONCLUSION

The digital version of the FAST showed strong psychometric properties in the general population sample, indicating that the instrument measures a multidimensional construct of functioning, encouraging its use by researchers and clinicians in their practice. Moreover, these findings would help to better understand the psychosocial implications of the pandemic and the importance of planning specific interventions to rehabilitee the affected group. Considering the previous reports [6], it can be concluded that mental health problems and poor psychosocial functioning may be a mark left by this pandemic.

## Figures and Tables

**Fig. (1):**
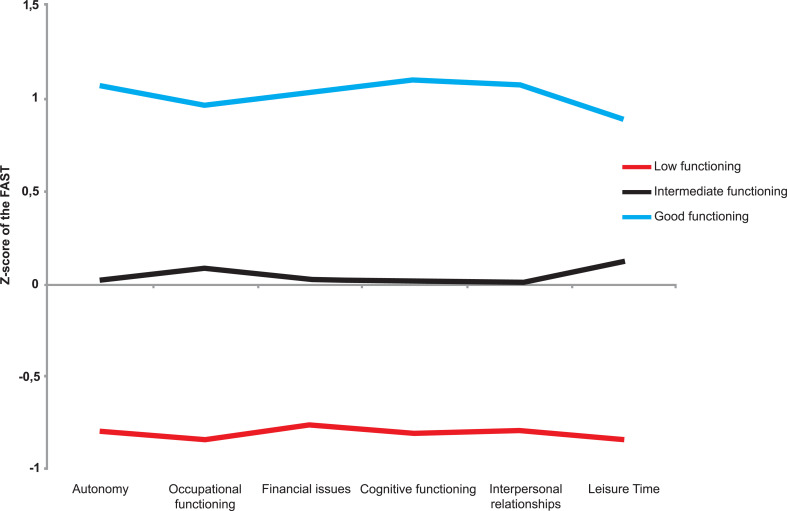
Mean of functioning performance between three clusters of individuals during COVID-19 pandemic. The X-axis is the functioning domains and Y-axis is the value of z-score based on mean and standard deviation of all sample.

**Fig. (2):**
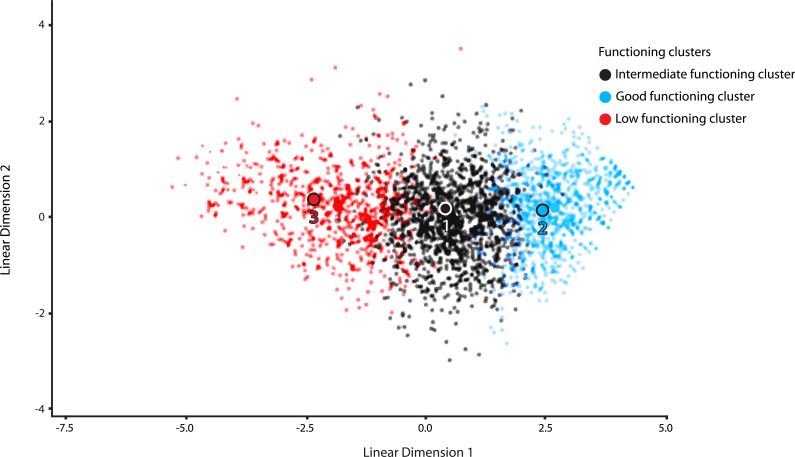
Discriminant validity: graphical agglomeration of the functional subgroups.

**Table 1:** Comparisons between the three functional clusters across overall and specific functioning domains of the FAST using one-way ANOVA with Tukey HSD post hoc test. Hedge’s g for a measure of effect size.

	**Low Functioning** **n= 661** **Mean(SD)**	**Intermediate Functioning** **n=1436** **Mean (SD)**	**Good Functioning** **n=926** **Mean (SD)**	**F-statistics**	**p-value**	**Hedge’s g** **(95%CI)** **Good vs. Low**	**Hedge’s g** **Good vs. intermediate**
Autonomy	6.01(2.60)^a^	3.14 (2.03)^b^	0.92 (1.41)^c^	1233.29	<.001	-2.5517 (-2.6853; -2.4181)	-1.2245 (-1.3142; -1.1348)
Occupational	9.70 (3.60)^a^	6.05 (3.33)^b^	2.19 (2.46)^c^	1106.60	<.001	-2.5122 (-2.6449;-2.3795)	-1.2782 (-1.3684;-1.1879)
Financial	3.94 (1.68)^a^	2.11 (1.46)^b^	0.67 (1.11)^c^	1034.82	<.001	-2.3747 (-2.5043;-2.2452)	-1.0793 (-1.1674; -0.9911)
Cognitive	8.98 (3.11)^a^	5.15 (2.58)^b^	2.29 (2.08)^c^	1305.18	<.001	-2.6123 (-2.7473;-2.4773)	-1.1930 (-1.2824; -1.1037)
Interpersonal	6.76 (2.68)^a^	3.67 (2.22)^b^	1.58 (1.82)^c^	1217.09	<.001	-2.3333 (-2.4620;-2.2046)	-1.0082 (-1.0956;-0.9207)
Leisure time	9.66 (1.93)^a^	6.89 (2.29)^b^	3.98 (2.44)^c^	981.95	<.001	-2.5325 (-2.6657; -2.3994)	-1.2379 (-1.3278; -1.1481)
FAST total	45.07 (8.05)^a^	27.00 (6.31)^b^	11.64 (5.61)^c^	5064.70	<.001	-4.9618 (-5.1612;-4.7623)	-2.5400 (-2.6499; -2.4301)

Note: Different letters mean difference between clusters: aLow Functioning *vs*. Intermediate Functioning; bIntermediate Functioning *vs*. Good Functioning; cGood Functioning *vs*. Low Functioning.

**Table 2:** Variables potentially affecting the three functional profiles.

**Characteristics**	**Good Functioning (n=926)** **No./mean**	**%/SD**	**Intermediate Functioning (n=1436)** **No./mean**	**%/SD**	**Low Functioning (n=661)** **No./mean**	**%/SD**	**F / χ2**	**p-value**
Age	38.5*	13.88	32.90^#^	11.48	31.35^§^	10.81	84.66	<.001
Sex, female n (%)^a^	737	79.8	1212	84.8	594	91.1	37.24	<.001
Work Situation							126.52	<.001
Employed	772	85.1	1219	85.1	483	73.1		
Unemployed	82	8.9	173	12.1	165	25		
Retired/Retired on disability	70	7.6	41	2.9	13	2		
Income (BRL)^b^							244.21	<.001
<708,19 - 2.965,69	207	22.4	498	34.7	369	55.8		
> 2.965,69 - 10.386,52	448	48.4	706	49.2	241	36.5		
> 10.386,52	271	29.3	232	16.2	51	7.7		
Occupation							29.00	<.001
Essential	312	33.7	406	28.3	141	21.3		
Non-essential	614	66.3	1030	71.7	520	78.7		
Previous psychiatric illness							146.44	<.001
Yes	264	28.5	598	41.6	389	31.1		
No	662	71.5	838	58.4	272	41.1		
Marital status							43.23	<.001
Married	488	52.7	594	41.4	249	37.7		
Single	438	47.3	842	58.6	412	62.3		
Education							70.16	<.001
Undergraduate	333	36	593	41.3	374	56.6		
Graduate/Postgraduate	593	64	843	58.7	287	43.4		
Impact of Event (IES-R)							640.46	<.001
Negative	839	90.6	977	68	198	30		
Positive	87	9.4	459	32	463	70		
Depression (PROMIS)							701.68	<.001
Moderate	322	34.8	1086	75.6	621	93.9		
Anxiety (PROMIS)							480.27	<.001
Moderate	581	62.7	1311	91.3	653	98.8		

Note: Different symbols mean difference between functioning conditions.

^a^ N=3005.

^b^ 1BRL= 0.574

## Data Availability

Data supporting the findings of the article is not publicly available as a result of the privacy policies of the health facilities involved in the study, but it can be provided by the corresponding author [A.R.R] upon reasonable request.

## LIST OF ABBREVIATIONS

WHO: World Health Organization
COVID-19: Severe Acute Respiratory Syndrome Coronavirus
FAST: Functioning Assessment Short Test
IES-R: Impact of Event Scale-Revised
PROMIS: Patient-Reported Outcomes Measurement Information System
CFA: Confirmatory Factor Analysis
KMO: Kaiser-Meyer-Olkin
PAM: Partition Around Medoids
